# Clinical characteristics and risk factors of preventable hospital readmissions within 30 days

**DOI:** 10.1038/s41598-021-99250-8

**Published:** 2021-10-11

**Authors:** Elsemieke A. I. M. Meurs, Carl E. H. Siegert, Elien Uitvlugt, Najla El Morabet, Ruth J. Stoffels, Dirk W. Schölvinck, Laura F. Taverne, Pim B. J. E. Hulshof, Hilde J. S. ten Horn, Philou C. W. Noordman, Josien van Es, Nicky van der Heijde, Meike H. van der Ree, Maurice A. A. J. van den Bosch, Fatma Karapinar-Çarkit

**Affiliations:** 1 Department of Clinical Pharmacy, OLVG, Noord-Holland, Amsterdam, The Netherlands; 2 Department of Internal Medicine, OLVG, Noord-Holland, Amsterdam, The Netherlands; 3 Department of Quality and Improvement, OLVG, Noord-Holland, Amsterdam, The Netherlands; 4 Department of Gastroenterology, OLVG, Noord-Holland, Amsterdam, The Netherlands; 5 Department of Cardiology, OLVG, Noord-Holland, Amsterdam, The Netherlands; 6 Department of Pulmonology, OLVG, Noord-Holland, Amsterdam, The Netherlands; 7 Department of Surgery, OLVG, Noord-Holland, Amsterdam, The Netherlands; 8 Department of Radiology, OLVG, Noord-Holland, Amsterdam, The Netherlands

**Keywords:** Health care, Risk factors

## Abstract

Knowledge regarding preventable hospital readmissions is scarce. Our aim was to compare the clinical characteristics of potentially preventable readmissions (PPRs) with non-PPRs. Additionally, we aimed to identify risk factors for PPRs. Our study included readmissions within 30 days after discharge from 1 of 7 hospital departments. Preventability was assessed by multidisciplinary meetings. Characteristics of the readmissions were collected and 23 risk factors were analyzed. Of the 1120 readmissions, 125 (11%) were PPRs. PPRs occurred equally among different departments (*p* = 0.21). 29.6% of PPRs were readmitted by a practitioner of a different medical specialty than the initial admission (IA) specialist. The PPR group had more readmissions within 7 days (PPR 54% vs. non-PPR 44%, *p* = 0.03). The median LOS was 1 day longer for PPRs (*p* = 0.16). Factors associated with PPR were higher age (*p* = 0.004), higher socio-economic status (*p* = 0.049), fewer prior hospital admissions (*p* = 0.004), and no outpatient visit prior to readmission (*p* = 0.025). This study found that PPRs can occur at any department in the hospital. There is not a single type of patient that can easily be pinpointed to be at risk of a PPR, probably due to the multifactorial nature of PPRs.

## Introduction

Unplanned readmissions are associated with higher morbidity and mortality and lower patient satisfaction^1^. In addition, readmitted patients demand hospital capacity and are a financial burden for healthcare institutions^2^. Therefore, various countries classify unplanned readmissions within 30 days as a performance indicator^3,4^. In 2012, the United States implemented the Hospital Readmission Reduction Program (HRRP) to shift the focus away from production-driven patient care. This program links payment of hospital care to quality outcomes and thus imposes penalties on hospitals that have excessive 30-day readmissions^5^. However, concerns remain on whether the HRRP is the correct parameter to use as a performance indicator^6^.

In addition to the concerns about using the correct parameter, a proportion of unplanned readmissions are due to the normal clinical course of patients and are therefore not preventable. Potentially preventable readmissions (PPRs) are a better performance indicator than the overall number of readmissions, since hospitals can act on preventing these PPRs^7^. A systematic review has identified median PPR rates of 27.1% of all readmissions, with a broad range varying from 5 to 79%^8^. Previous studies explored different mechanisms leading to PPRs, including management-related, medication-related, and diagnostics-related causes^9,10^.

Heterogeneity exists between the studies that evaluated PPRs, and conflicting risk factors were identified^10–16^. This can be due to a different assessment of preventability per study, as it is either automated by an algorithm or done manually by physicians, resulting in different classification of PPRs^17,18^. In addition, studies generally focus on only 1 specialty, which could result in missing PPRs that occur across departments^18^.

Evidence is lacking on the difference in characteristics between PPRs and non-PPRs, such as readmissions across departments, differences in lengths of stay, and critical care need during readmission. Gaining more understanding of the readmission characteristics and the risk factors that lead to PPRs could create opportunities for interventions targeted at high-risk patients.

Therefore, the primary aim of this study was to analyze the clinical characteristics of PPRs compared to non-PPRs. Secondly, we aimed to identify risk factors specifically for PPRs. PPRs were identified by means of a multidisciplinary review of the readmissions.

## Methods

### Study population and design

This study is a cross-sectional single-center observational study at an urban teaching hospital, OLVG in Amsterdam, the Netherlands. Patients were prospectively included from July 2016 to February 2018. The study was approved by the local scientific review board of the hospital (“Advies Commissie Wetenschappelijk Onderzoek Medische Ethische Commissie,” ACWO-MEC; registration number 16-028). As this study does not influence the patient’s integrity, the need for informed consent was waived by the scientific review board (ACWO-MEC) of the hospital (according to Dutch legislation). The study methods were performed in accordance with relevant guidelines and regulations. Patient data were obtained and processed in accordance with national privacy regulations. This study was performed in accordance with the STROBE guidelines.

All 30-day readmissions at our center were identified by means of an algorithm in the electronic health record system. These readmissions were screened daily by the study coordinator, a medical doctor, for inclusion eligibility. Inclusion criteria were unplanned readmissions within 30 days after discharge from 1 of 7 participating departments: Internal Medicine, Surgery, Cardiology, Pulmonology, Neurology, Gastroenterology, and Psychiatry. These departments were chosen based on their relatively high readmissions rates in previous years. Exclusion criteria were patients younger than 18 years, initial admission (IA) that was a short stay, patients transferred to another hospital during IA, and patients who left the hospital against medical advice. Cases were also excluded if a readmission was clearly not related to the initial admission (e.g., pneumonia followed by a car accident without any link to the initial admission). Both the study coordinator and the initial admission residents assessed whether the readmission was related to the care provided during the initial admission.

### Assessment of preventability

The study methodology has been published before; however, the previous study primarily focused on the method of assessing preventability and analyzed the causes of PPRs in a smaller cohort of patients^9^. In summary, to distinguish between PPR and non-PPR, the readmissions were manually reviewed by physicians and pharmacists in 2 phases. In the first phase, each readmission was reviewed separately by a resident of the initial admission department and by a hospital pharmacy resident, using a specifically designed reviewing tool. A readmission received a causality score and, if applicable, a preventability score that ranged from 1 to 6. If the causality score was 4 or higher, the preventability of the case was scored. A readmission was rated preventable if actions could have been taken within the scope of the hospital. Scores from 1 to 3 were considered not preventable and scores from 4 to 6 were considered preventable. This scoring system is similar to those cited in the previous literature^10,19^.

The second phase of the reviewing process consisted of multidisciplinary meetings, which were held with residents of all 7 included departments, including hospital pharmacists. A readmission was discussed in a multidisciplinary meeting if the resident from the initial department or the resident of the hospital pharmacy rated a case either preventable or if the study coordinator considered a case atypical. During the meetings, the readmissions received a final preventability score. If no consensus was reached on a preventability score, a senior professional was consulted. To minimalize interpretation bias, all residents received extensive training and instructions prior to reviewing cases. A subset of the reviewed readmissions was validated by 2 blinded experienced senior reviewers, a clinical pharmacologist, and an internal medicine physician. This validation showed no big differences between the assignment of preventability by residents compared to the assignment of preventability by the senior reviewers. The Cohen’s Kappa Statistic agreement was 0.78.

### Data collection and outcomes

Data was collected by extracting information from the electronic patient health record system. A protocol was developed to assess unstructured data (e.g., living situation or language barrier). Subsets of data that were manually collected by students were checked by the study coordinator to reduce the amount of collection bias. Data extracted from the hospital information system was manually validated to get insights in potential registration errors.

Data that were collected included patient characteristics, social factors, medical history, initial admission (IA) characteristics, IA discharge features, and follow-up data. Previous studies that assessed different risk-factors for general readmissions and preventable readmissions were analysed^11^ and important potential risk factors were selected for the inclusion in our study, if they were available in the EHR.

The socio-economic status (SES) of patients was identified by their postal codes and is a combined measure of 4 variables provided by the Dutch national institute SCP (the Netherlands Institute for Social Research). The updated Charlson comorbidity index (CCI) score was based on ICD-10 codes generated by the hospital information system^20^. A patient was considered to have an oncology disease if there was any form of treatment or hospital visit related to an oncology disease a year before the initial admission. Previous clinical admissions and emergency department visits were included if they took place within 6 months prior to the initial admission. Every unique ward a patient was admitted to during initial admission, including the intensive care unit (ICU), was considered a unique department. Changed living situation was scored “yes” when a patient moved to a new resident type after IA discharge. Decreased cognition was scored “yes”, when the EHR contained comments suggesting cognitive impairment, like the terms ‘cognition,’ ‘memory,’ ‘forgetfulness’ and when there were presumptions of cognitive impairment noted in the EHR. Last, the medical history of the patient was screened for cognitive impairment. Outpatient clinic visit before readmission was scored “yes” if any outpatient visit between IA and readmission was attended.

Data that were collected to analyze clinical characteristics of the readmissions included: days between discharge and readmission, length of hospital stay, the medical specialty of the specialist during IA, readmission by a practitioner of the same specialty as IA, ICU visit, and in-hospital mortality. We considered a readmission within 7 days after discharge an early readmission^19^.

### Statistical analysis

Normally distributed continuous variables were analyzed with one sample T-tests and are reported using means and standard deviation (SD). Non-normal distributed continuous variables were analyzed with Mann–Whitney U tests and are reported using medians and interquartile range (IQR). Categorical variables were analyzed with chi-square test and are reported as total numbers and percentages. For analyzing associations of potential risk factors with PPR, generalized estimation equation (GEE) models were fitted using a logistic model to estimate odd ratios with 95% confidence intervals. First, univariable models for potentials risk factors were fitted separately to estimate unadjusted odds ratios. Potential risk factors with a *p* value of < 0.20 in the univariable analyzis were selected for the adjusted analysis procedure^10,21^. We selected a p-value of 0.20 because we had many factors and did not want to exclude important factors^21^. For these risk factors, multivariable models were adjusted for the other potential risk factors to control for confounding. Stepwise forward selection was applied in sequence of lowest p-value to highest p-value. P-values lower than 0.05 were considered statistically significant. All data were analyzed with R (version 4.0.3, package “geepack”^22^).

## Results

**Figure 1:**
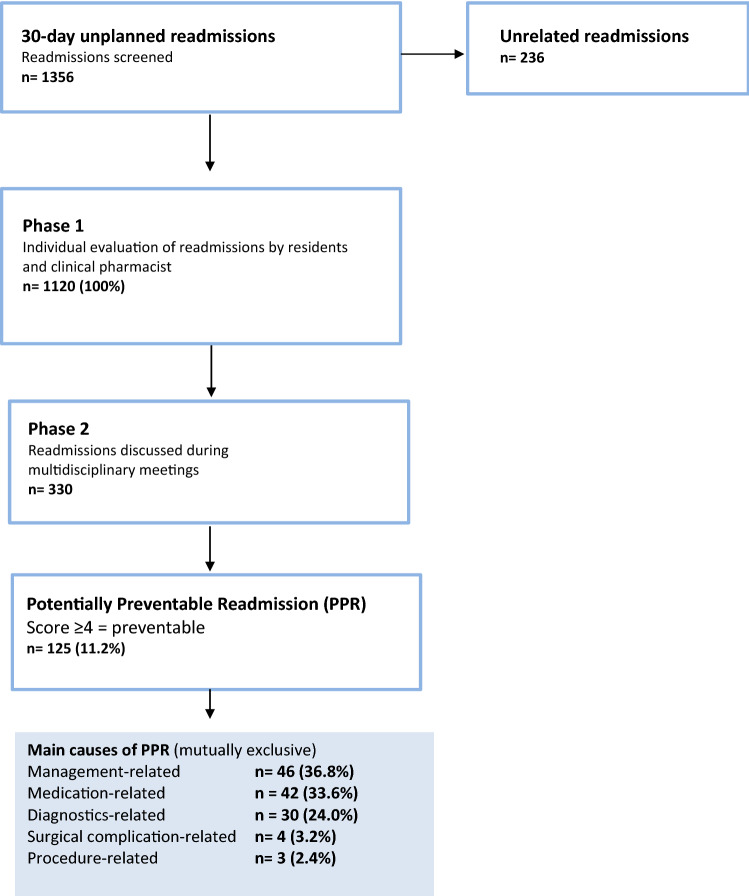
Flowchart readmission inclusion and final preventability scores.

We identified 1356 unplanned readmissions from 7 departments. Of these readmissions, 236 did not meet the inclusion criteria because they were unrelated to the initial admission. The remaining 1120 readmissions were included in the study, consisting of 878 patients. Of the readmissions, 125 (11%) were PPRs. Figure 1 shows that 37% of the PPRs were management-related, 34% were medication-related, and 24% were diagnostic-related.

Table 1 shows the patient demographics and initial admission characteristics. Males and females were equally represented in the cohort (male 49.6%). The mean age in the study was 64.2 years, and 18.6% of the total cohort had decreased cognition. Prior to IA, 34.3% of the patients lived alone and 7.5% lived in an institution. The median length of stay was 5 days.

**Table 1:** Patient demographics and initial admission (IA) characteristics.

	Total cohort n = 1120	PPR n = 125	Non-PPR n = 995	*p* value
**Patient characteristics**				
Gender, male, n (%)	556 (49.6%)	63 (50.4%)	493 (49.5%)	*p* = 0.86
Age, mean ± SD	64.2 ± 17.0	68.0 ± 16.1	63.7** ± **17.1	*p* = 0.01
**Social factors**				
Language barrier present, n (%)	229 (20.4%)	21 (16.8%)	208 (20.9%)	*p* = 0.28
Decreased cognition, n (%)	208 (18.6%)	23 (18.4%)	185 (18.6%)	*p* = 0.96
Social Economic status, median (IQR)	− 0.41 (− 1.26–0.47)	− 0.31 (− 1.08–0.80)	− 0.41 (− 1.26–0.47)	*p* = 0.08
Living situation, n (%)				*p* = 0.63
With partner at home	617 (56.9%)	65 (52.8%)	552 (57.4%)	
Alone at home	384 (35.4%)	48 (39.0%)	336 (34.9%)	
Institution	84 (7.7%)	10 (8.1%)	74 (7.7%)	
**Medical history**				
CCI score, median (IQR)	1 (0–2)	1 (0–2)	1 (0–2)	*p* = 0.26
Oncology, n (%)	247 (22.1%)	23 (18.4%)	224 (22.5%)	*p* = 0.30
Palliative care, n (%)	113 (10.1%)	11 (8.8%)	102 (10.3%)	*p* = 0.64
GFR < 50, n (%)	221 (20.0%)	29 (23.4%)	192 (19.6%)	*p* = 0.31
Previous hospital admission (< 6 months), n (%)	518 (46.3%)	46 (36.8%)	472 (47.4%)	*p* = 0.02
Number of admissions (< 6 months), mean ± SD	0.9 ± 1.4	0.6 ± 1.1	1.0 ± 1.5	*p* = 0.01
Previous ED visit (< 6 months), n (%)	284 (25.4%)	23 (18.4%)	261 (26.2%)	*p* = 0.06
**Admissions characteristics**				
Planned admission, n (%)	223 (19.9%)	21 (16.8%)	202 (20.3%)	*p* = 0.36
Length of stay, days, median (IQR)	5 (2–9)	5 (2–10)	5 (2–9)	*p* = 0.58
Unique departments during IA, median (IQR)	1 (1–2)	2 (1–2)	1 (1–2)	*p* = 0.06
ICU admission, n (%)	88 (7.9%)	13 (10.4%)	75 (7.5%)	*p* = 0.26
Number of consultations during IA, median (IQR)	0 (0–1)	0 (0–1)	0 (0–1)	*p* = 0.94
Consultation by readmission specialty during IA, n (%)	67 (6.0%)	8 (6.4%)	59(5.9%)	*p* = 0.83
**Discharge characteristics**				
Changed living situation, n (%)	67 (6.0%)	9 (7.2%)	58 (5.8%)	*p* = 0.55
Discharge to home, n (%)	986 (88.0%)	107 (85.6%)	879 (88.3%)	*p* = 0.37
Discharge in weekend, n (%)	176 (15.7%)	19 (15.2%)	157 (15.8%)	*p* = 0.87
Discharge letter send before readmission, n (%)	594 (53.0%)	67 (53.6%)	527 (53.0%)	*p* = 0.89
Discharge letter send ≤ 1 day after discharge, n (%)	234 (20.9%)	29 (23.2%)	205 (20.6%)	*p* = 0.50
Number of medication at discharge IA, Median (IQR)	10 (5–14)	10 (6–14)	10 (5–14)	*p* = 0.56
Medication at discharge ≥ 5, n (%)	888 (79.5%)	105 (84%)	783 (79%)	*p* = 0.21
Number of medication changes, median (IQR)	2 (1–4)	3 (1–5)	2 (1–4)	*p* = 0.36
Medication changes during admission ≥ 5, n (%)	232 (20.7%)	34 (27.2%)	198 (20.0%)	*p* = 0.08
**Follow-up**				
Outpatient clinic planned, n (%)	956 (85.4%)	106(84.8%)	850 (85.5%)	*p* = 0.83
Outpatient clinic visited before readmission, n (%)	423 (37.8%)	36(28.8%)	387 (38.9%)	*p* = 0.03

IA = initial admission, CCI = Charlson Comorbidity index, PPR = potentially preventable readmissions.

Table 2 summarizes the readmission features of PPR vs. non-PPR. There were significantly more early readmissions in the PPR group (PPR 54.4% vs. non-PPR 44.2%, *p* = 0.03). Additionally, in the PPR group, the period between discharge and readmission was 3 days shorter (median, PPR 6 days vs. non-PPR 9 days, *p* = 0.04). In the PPR group the length of stay was longer; however, this difference was not statistically significant (median PPR 6 days vs. non-PPR 5 days, *p* = 0.16). PPRs occurred from all 7 medical specialties and no significant differences were found among these specialties (*p* = 0.21). In the PPR group, patients were more often readmitted by another medical specialty (PPRs 29.6% vs. non-PPRs 21.7%, *p* = 0.07). There was no significant difference in number of deaths and ICU admissions between both groups, even though the number of ICU admissions was higher in the PPR group (PPR 11% vs. non-PPR 6%, *p* = 0.08).

**Table 2:** Readmission characteristics of PPRs vs. non-PPRs.

Readmission characteristics	PPR n = 125	Non-PPR n = 995	*p* value
Days between discharge and readmission			
median (IQR)	6 (2–13)	9 (4–17)	*p* = 0.04
Early readmission (≤ 7 days), n (%)	68 (54.4%)	440 (44.2%)	*p* = 0.03
LOS readmission, days, median (IQR)	6 (3–13)	5 (3–10)	*p* = 0.16
Readmission after IA from department, n (%)			*p* = 0.21
Surgery	36 (28.8%)	250 (25.1%)	
Cardiology	23 (18.4)	124 (12.5%)	
Internal Medicine	31 (24.8%)	229 (23.0%)	
MDL	13 (10.4%)	131 (13.2%)	
Lung	16 (12.8%)	210 (21.1%)	
Neurology	5 (4.0%)	40 (4.0%)	
Psychiatry	1 (0.8%)	11(1.1%)	
Different specialty than initial admission, n (%)	37 (29.6%)	216 (21.7%)	*p* = 0.07
ICU admission during readmission, n (%)	14 (11.2%)	60 (6.0%)	*p* = 0.08
Died during readmission, n (%)	7 (5.6%)	53 (5.3%)	*p* = 0.90

IA = initial admission, LOS = length of stay, PPR = potentially preventable readmissions. *p* values are adjusted for repeated patients in the cohort by means of GEE-analysis.

We analyzed 23 risk factors, of which 7 were included in the adjusted multivariate analysis. Table 3 presents 4 factors that differed significantly in the adjusted analysis between PPRs and non-PPRs. More hospital admissions during the preceding 6 months decreased the odds of a PPR (adjusted OR = 0.77; *p* = 0.004). Second, higher age significantly increased the odds of PPR (adjusted OR = 1.02; *p* = 0.004). Third, a higher socio-economic status score increased the odds of a PPR (adjusted OR = 1.17; *p* = 0.049). Finally, patients who visited the outpatient clinic prior to readmission had lower odds of a PPR (adjusted OR = 0.63; *p* = 0.025). Other potential risk-factors, like the Charlson comorbidity index score, were not significantly associated with PPRs.

**Table 3:** Univariable and multivariable association among potential risk factors and potentially preventable readmission (PPRs).

Risk factors for PPRs	Unadjusted OR (95% CI)	*p* value	Adjusted OR (95% CI)	*p* value
Number of previous hospital admissions < 6 months	0.80 (0.67–0.94)	0.009	0.77 (0.65–0.92)*	0.004
Age (years)	1.02 (1.00–1.03)	0.010	1.02 (1.01–1.03)^†^	0.004
Outpatient clinic visited before readmission (yes)	0.63 (0.42–0.94)	0.025	0.63 (0.42–0.94)^‡^	0.025
Socio-economic status score	1.17 (1.00–1.36)	0.049	1.17 (1.00–1.36)^‡^	0.049
Previous ED admissions < 6 months (yes)	0.64 (0.40–1.02)	0.059	0.76 (0.47–1.23)*^†^	0.264
Number of unique departments during IA	1.19 (0.96–1.46)	0.110	1.07 (0.86–1.35)*^†§^	0.539
Number of medication changes during IA	1.05 (0.98–1.13)	0.180	1.03 (0.95–1.12)*^†^¶^	0.518

ED = Emergency Department, IA = initial admission, SES = Socio-economic status.

Adjusted for: * = age; ^†^ = Previous hospital admissions < 6 months; ^^^ = SES score; ^¶^ = Number of unique departments during IA; ^§^ = Number of medication changes during IA; ^‡^ = Unadjusted analysis, no confounding factors found during step-wise selection.

## Discussion

With this study we aimed to gain insights into the differences in clinical characteristics of PPRs compared to non-PPRs. Our results indicate that these differences exist, including the time until readmission and the readmitting medical specialty. Second, our goal was to identify variables that are associated with preventable readmissions to gain more insight into which patients should be targeted to reduce preventable readmissions. To our knowledge, this study comprised the largest cohort of multidisciplinary evaluated readmissions to date, including patients who were admitted by a broad variety of medical specialties.

We found that PPRs occurred equally between the different medical specialties, illustrating the importance of a broad hospital approach when analyzing and reducing PPRs. In contrast, previous studies analyzing PPRs focused mostly on a subset of patients instead of including patients of all specialties^10,15^, possibly focusing on the few diagnoses that the Hospital Readmission Reduction Program (HRRP) targets^5^. This focus, however, fails to identify that PPRs are a hospital-wide problem that affects more than just a select group of patients.

This need for a hospital-wide approach is strengthened by our results showing that 30% of the PPRs were readmitted by a practitioner with a different specialty than the IA practitioner. This finding is interesting because it shows that a large portion of PPRs are due to a new problem requiring readmission through a new medical specialty. Interestingly, 81% of previous studies evaluating PPRs did not include these all-cause readmissions, potentially missing a large portion of cases^18^.

Our results indicate that early readmissions are more likely to be preventable than late readmissions. This is in line with other studies^15,19^. Grahem et al. found that early readmissions are related to physician decision-making regarding diagnosis and management matters in the hospital. They found that for these early readmissions, in 47% of cases the hospital was the ideal location for intervention to prevent the readmission^19^. This suggests we should focus on early readmissions when creating hospital-focused programs to reduce readmissions.

Our results showed that PPRs had fewer hospital admissions in the preceding 6 months than did non-PPRs. This was an unexpected outcome; however, our hypothesis is that non-PPR patients are generally sicker than patients with fewer hospital admissions. Therefore, the readmission is possibly often due to progression of the disease. However, further studies are needed to fully understand this unexpected finding.

Risk factors for all types of readmissions have been extensively investigated^11^; however, they cannot be directly applied for PPRs. We analyzed numerous potential risk factors, of which only 4 were significantly associated with PPRs. The few studies that analyzed potential risk factors specifically for PPRs did not show consistency in results either^10–14,23^. The lack of clear intervenable risk factors in this analysis and in previous studies suggests that any patient can have PPRs and that patients at risk for them cannot easily be pinpointed. Conversely, possibly important discriminating factors are not registered during patients’ clinical course; those include the presence of a social network, patients’ health literacy, experience of the medical doctors, and the workload at a department at a specific time point.

Compared to the previous literature, our study stands out in 3 different ways. First, manually reviewing preventability has been the preferred method^24^, especially when multiple reviewers are involved^25^. To our knowledge, this is the largest cohort of patients that were manually reviewed and where preventability was assessed in a multidisciplinary way, reducing interpretation bias. This was important, since inter-reviewer differences occur when manually reviewing cases^26^. Second, we included readmissions after discharge from 7 different medical specialties, making the study more generalizable. We included all-cause readmissions, among them readmissions to specialty other than the specialty of the IA physician. This prevents underestimation of the true number of readmissions. Third, many potential risk factors for readmissions were analyzed, including variables from different origin like social factors, previous medical history, and follow-up data.

However, this study merits some consideration. First, retrospectively assessing preventability could result in documentation and hindsight bias. We included patients prospectively, though, and tried to minimize the time until review of the readmission. Hindsight bias was actively discussed, and in case of doubts the supervisors were consulted. Second, we were limited by the data that was available in the medical records. This could lead to missing information, like medical histories that were incompletely collected during hospitalization and which were used to calculate the CCI. SES was calculated using patients’ postal codes, creating an aggregated data point, possibly not reflecting the scores of individual patients. Additionally, important potential risk factors are neither regularly assessed nor documented in hospitals. For instance, often cognitive impairment^27^ is not fully documented in the EHR and we were missing important integrated care-related factors, and these have also not been addressed in other work focusing on PPR^18^. Third, this is a mono-center study, limiting external generalizability. Also, readmissions to other hospitals were not included in the cohort. A previous Dutch study showed only 8.9% of readmissions taking place in another hospital^28^, so we believe this small percentage did not influence the results. Finally, although we had an extensive review method for readmissions, there is still a possibility that some PPRs were missed during the evaluation process. However, the validation by senior reviewers did not differ widely from the study.

Future work should include readmissions of all hospital specialties, evaluating all-cause readmissions. Additionally, they should include factors that are not routinely documented in clinical settings, but which are important for the continuing of good patient care and affect readmissions. Examples of these important factors include structured documentation of cognitive impairment index^29,30^, health literacy of patients, frailty scores^31^, and social environment factors^32–35^. Also, the points of view of primary care providers and patients should ideally be included^36^, especially as there is evidence that readmissions after 7 days could be prevented in the non-hospital environment^19^.

Our large all-cause readmission study demonstrated that PPRs can occur in any department and the patients are quite often readmitted by a practitioner of a different medical specialty. Additionally, PPRs are more often early readmissions than are non-PPRs. There is not one type of patient that can easily be pinpointed to be at risk of a PPR. This might be because PPRs are caused by a complex chain of events that start during initial admission but proceed after hospital discharge. Further studies should prospectively include factors not routinely collected when evaluating PPRs and determine whether these factors correlate to PPRs. Since the goal is to prevent readmissions, it is important to accurately identify the patients for whom the readmission is truly preventable.

## Data Availability

Data are available from the authors upon reasonable request and with permission.
